# Supplementary material for: Generation of out-of-plane polarized spin current by spin swapping
Source: Nat Commun. 2023 Jul 28;14:4549. doi: 10.1038/s41467-023-39884-6 (PMC10382594; doi:10.1038/s41467-023-39884-6)
Supplement: Supplementary file 1 — Supplementary Information [file 41467_2023_39884_MOESM1_ESM.pdf]

Supplementary Information

# Generation of out-of-plane polarized spin current by spin swapping

Binoy K. Hazra[1,†], Banabir Pal[1,†], Jae-Chun Jeon[1], Robin R. Neumann[2], Börge Göbel[2], Bharat Grover[1], Hakan Deniz[1], Andriy Styervoyedov[1], Holger Meyerheim[1], Ingrid Mertig[2], See-Hun Yang[1], Stuart S. P. Parkin[1,*]

[1]Max Planck Institute of Microstructure Physics, Weinberg 2, 06120 Halle (Saale), Germany

[2]Institut für Physik, Martin-Luther-Universität Halle-Wittenberg, D-06099 Halle (Saale), Germany

[†]These authors contributed equally to this work.

*To whom correspondence should be addressed: stuart.parkin@halle-mpi.mpg.de

**This PDF file includes:**

Supplementary Text

Figs. S1 to S18

References

## I. Structural analysis of $Mn_3Sn$/Py, Cu/Py, Ru/Py, Re/Py and Pt/Py films

To analyze the crystal symmetry of $Mn_3Sn$, X-ray diffraction experiments were carried out by using a Gallium-Jet X-ray source operated at 70 keV and 100 W emitting Ga-$K_\alpha$ radiation ($\lambda$=1.3414 Å) monochromatized and focused onto the sample by a Montel optics providing a highly collimated beam of 100 μm in height and 2 mm in width. For data collection a six-circle x-ray diffractometer operated in the z-axis mode[1] was used, where the incident beam was kept constant at an angle of $\mu$=2° to enhance the scattered intensity of the 12 nm thick $Mn_3Sn$ film by simultaneously penetrating the 5 nm Py and 3 nm TaN layers. Integrated reflection intensities were measured by rotating the sample around the surface normal (theta-scan) while the position of the 2-dimensional (2D) pixel detector was kept fixed at an in-plane ($\delta$) and out-of-plane ($\gamma$) angle given for each reflection (HKIL)[1].

The data set for $Mn_3Sn$ film consists of about 22 reflections which reduce to 12 symmetry independent reflections after averaging over symmetry equivalent reflections based on the 6mm symmetry of the (0001) sample surface. Subsequently, the observed structure factor magnitudes, $|F(HKL)_{obs}|$, were derived from the integrated intensities by multiplying with instrumental factors (Lorentz, polarization- and effective area)[2-3]. In the first step of the structure analysis, the bulk structure model was considered to fit the calculated $|F(HKL)_{calc}|$ to the observed ones $|F(HKL)_{obs}|$. The structure model is shown in Fig. 1(a) in projection along the [0001] axis (main text). Bulk $Mn_3Sn$ is reported to crystallize in the inversion symmetric space group (SGR) $P6_3/mmc$ (PDF 01-073-2857)[4], in which Sn and Mn atoms occupy Wyckoff sites 2d ($^1/_3$, $^2/_3$, ¾) and 6h (x, 2x, $^1/_4$), respectively. Owing to the high symmetry of the structure, the only free parameter is the x-position of the Mn-atoms and the atomic displacement parameters (ADP) of the Sn- and Mn atoms[5]. All observed reflections are in agreement with the general condition for their appearance [(H H $2\overline{H}$ L): L=2n, with n integer].

In the first step of the quantitative analysis the calculated |F(HKL)| were fitted to the experimental ones based on SGR P6$_3$/mmc using the least-squares refinement based on non-weighted refinement which minimizes the non-weighted residuum ($R_{\mathrm{u}}$) given by: $R_{\mathrm{u}} = \frac{\sum_{HKL} ||F(HKL)_{obs}|-|F(HKL)_{clac}||}{\sum_{HKL} |F(HKL)_{obs}|}$, where the summation runs over all reflections (HKL). For the $Mn_3Sn$ film, we derive $R_{\mathrm{u}}$=0.190 (see Table 1).

| $R_{\mathrm{u}}$=0.194 | Sn (2d) | Mn (6h) |
|---|---|---|
| Θ | 1.00 | 1.00 |
| x | 0.333 | 0.355 |
| y | 0.666 | 0.177 |
| z | 0.750 | 0.750 |
| $U^{11}$ (Å$^2$) | 0.010 | 0.023 |
| U33 (Å$^2$) | 0.030 | 0.070 |

Table 1: Structure parameters derived from least-squares fit for the 12 nm $Mn_3Sn$ film based on the SGR P6$_3$/mmc. Atomic positions are given in relative units with respect to the lattice parameters a=b=5.67 Å, c= 4.53Å. Uncertainties are in the order of 1-2%.

In the next step, the Fourier-Transform (FT) of the structure factors was calculated to derive the three-dimensional charge density [ρ(x,y,z)] within the unit cell, which is given by: $\varrho(\mathrm{x,y,z}) = \sum_{HKL} \pm|F(HKL)_{obs}| \cos 2\pi\ (H\mathrm{x} + K\mathrm{y} + L\mathrm{z})$. Since the structure models are inversion-symmetric the experimental structure factors as coefficients of the Fourier series are real numbers. Thus, only the signs in front of the structure factor magnitudes [F(HKL)= ± |F(HKL)|] are needed for the calculation. They are calculated based on the structure model. As long as its gross features are correct (as in the present case) the FT is able to provide a direct view of the structure and helps to identify unknown details. In Fig. S1(a) the charge density of the 12 nm film is shown in projection along [0001]. In comparison with the bulk structure

model in Fig. 1(a) (main text), it is directly evident that the intense maxima are linked to the Sn and Mn-atoms. The distinct difference between the samples is that [Fig. S1(a)] there is another maximum at the origin of the unit cell (x,y)=(0,0) (see arrow).

The structure refinement involving a fractional occupation of the site labelled by the Wyckoff notation (2b) in SGR $P6_3/mmc$ dramatically improved the fit as shown by the contour plot in Fig. S1(b). We emphasize that there is another faint charge density at $(^1/_2, ^1/_2, z)$, which however does not lead to a significant improvement in the fit quality.

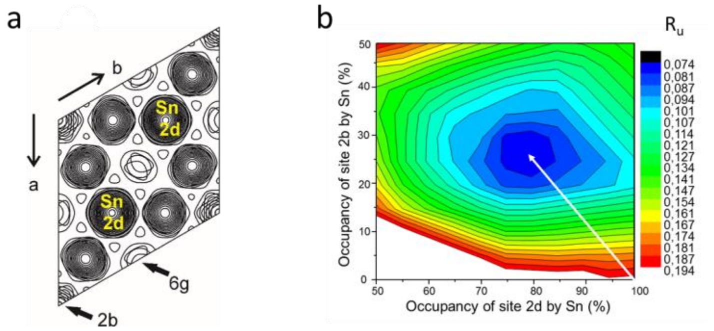


**Fig. S1. Structural characterization of $Mn_3Sn$.** (a) Charge density of the 12 nm $Mn_3Sn$ film projected along [0001]. The charge density of the Sn atoms is labelled. Note the additional charge density near (0,0) and $(^1/_2,0)$ as indicated by the arrows. (b) Contour plot of $R_u$ versus site occupancy of the positions 2d and 2b by Sn in SGR $P6_3/mmc$. About 25% of Sn atoms are located at 2b while vacancies are created at 2d. The fit quality dramatically improves from about $R_u$=0.19 for the perfectly ordered structure to $R_u$=0.07 for the disordered one (see arrow). The uncertainty of the site occupancy is about 5 percentage points.

Ru(5 nm)/Py(5 nm) and Re(5 nm)/Py(5 nm) films are grown at room temperature on $Al_2O_3$ (0001) substrate. Out-of-plane X-ray diffraction confirms the epitaxial nature of all the bilayers as shown in Fig. S2(a-b). On the other hand, Cu(5 nm)/Py(5 nm) and Pt(5 nm)/Py(5 nm) bilayers are sputtered on both Si (001)/$SiO_2$ (25 nm) and $Al_2O_3$ (0001) substrates at room temperature. It can be seen that the crystallinity of Pt/Py is better on $Al_2O_3$ substrate compared to Si/$SiO_2$ substrate (Fig. S3(c,d)) whereas Cu/Py grows amorphous and epitaxial on Si/$SiO_2$ and $Al_2O_3$ substrates (Fig. S3(a,b)), respectively. The surface roughness is found to be less than

0.5 nm for all the $Mn_3Sn$/Py and other bilayers. A typical atomic force micrograph is displayed in Fig. S4(a) for $Mn_3Sn$/Py. Further cross-sectional transmission microscopy reveals that $Mn_3Sn$ is well-ordered and confirms the thicknesses of all the layers (Fig. S4(b)).

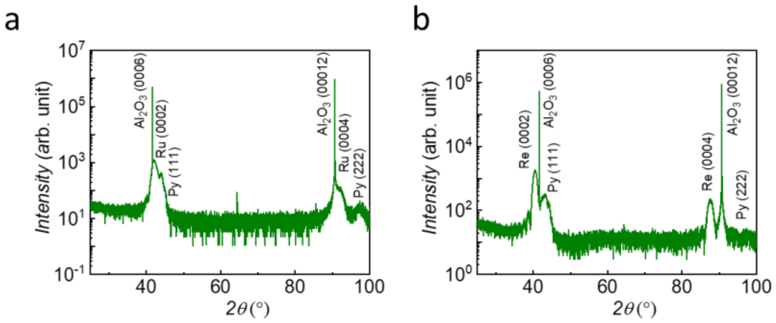


**Fig. S2. Structural characterization of Ru and Re.** (a-b) X-ray diffraction patterns of Ru/Py and Re/Py bilayers.

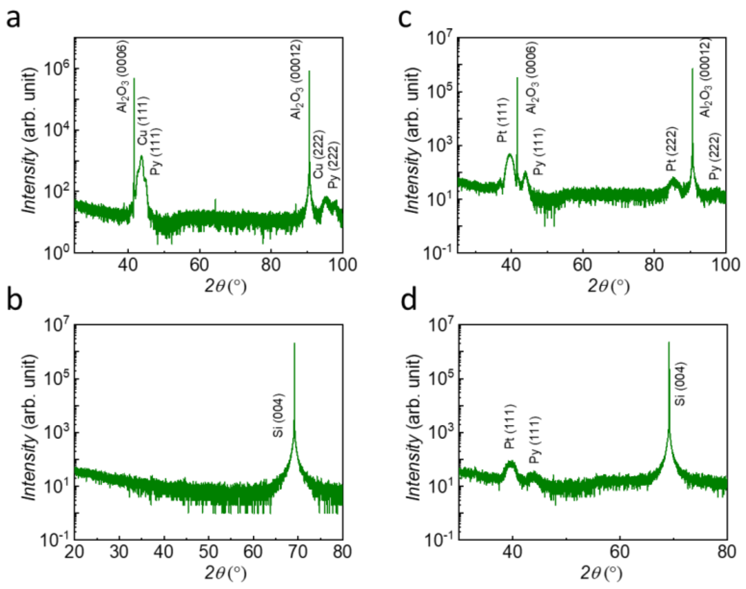


**Fig. S3. Structural characterization of Pt.** X-ray diffraction patterns of (a-b) Cu/Py and (c-d) Pt/Py bilayers which are grown on $Al_2O_3$ (0001) and Si (001)/$SiO_2$ substrates, respectively.

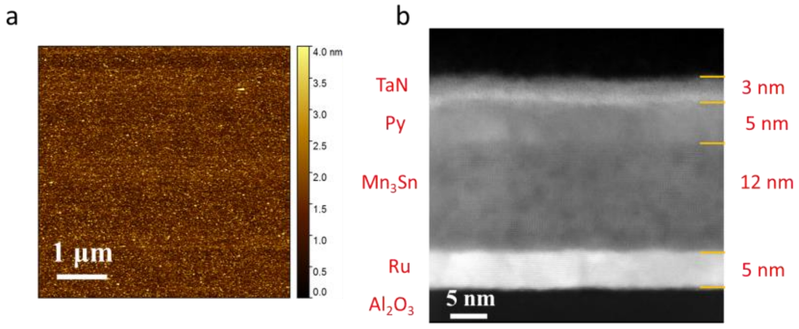


**Fig. S4. AFM and STEM characterization of $Mn_3Sn$**. (a) Atomic force micrograph of the $Mn_3Sn$(12 nm)/Py(5 nm) thin film. (b) Scanning transmission electron micrograph of the same film.

**II. Magnetic properties of $Mn_3Sn$ and exchange bias in $Mn_3Sn$/Py thin films**

Magnetization ($M$) as a function of in-plane magnetic field ($H_{\parallel}$) has been measured on a single layer of 12 nm $Mn_3Sn$ (0001) film to probe the small in-plane moment which lies in the kagome plane. From the $M$ vs. $H_{\parallel}$ data, a small in-plane magnetization (~20 emu/cm$^3$) at 300 K is observed when the diamagnetic contribution is subtracted from the substrate (Fig. S5(a)). The small magnetization is consistent with the previous report on the thin film[6]. Further, magnetization decreases with an increase in temperature which indicates the Néel temperature beyond 400 K (Inset of Fig. S5(a)). A detailed exchange bias (EB) measurement has been performed on the $Mn_3Sn$/Py thin films to confirm the antiferromagnetic structure of $Mn_3Sn$. The $Mn_3Sn$/Py film was cooled from 400 K to the measurement temperature in the presence of a negative in-plane magnetic field ($H_{\parallel} = -10$ kOe) and magnetic hysteresis was recorded at the respective temperatures. A large shift of the magnetic hysteresis loop along the positive magnetic field axis has been observed (inset of Fig. S5(b)) at 5 K. This shift decreases with an increase in the temperature and a symmetric hysteresis (i.e., EB vanishes) has been observed at 280 K which is the blocking temperature ($T_B$) for the $Mn_3Sn$ (12 nm)/Py (5 nm) film (Fig. S5(b)). The blocking temperature decreases systematically with a decrease in $Mn_3Sn$ layer thickness (Fig. S5(c)). Note that since the $T_B$ is less than 300 K for all the bilayers, EB does not affect the SOT measurements at 300 K.

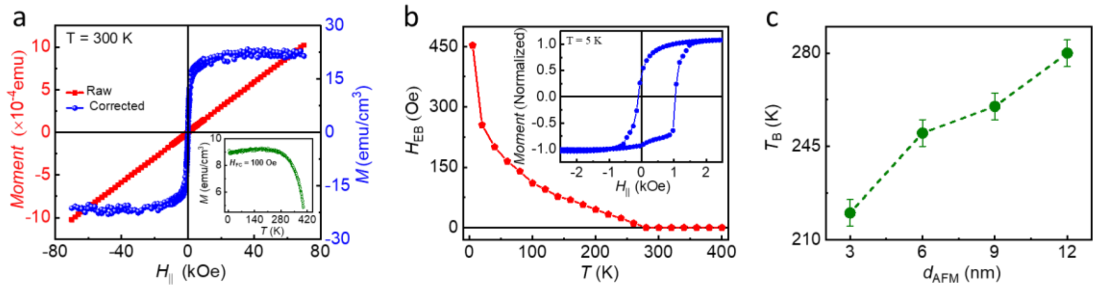


**Fig. S5. Magnetic properties of $Mn_3Sn$ and $Mn_3Sn$/Py.** (a) Raw and corrected $M$ vs. $H_{\|}$ for the 12 nm single layer $Mn_3Sn$ film. Inset shows the temperature dependence of magnetization for the same film measured in field-cooled ($H_{FC}$=100 Oe) condition. (b) EB field as a function of $T$ for the $Mn_3Sn$(12 nm)/Py(5 nm) film. Inset shows a representative $M$ vs. $H_{\|}$ measured at 5 K after field-cooled. (c) $T_B$ as a function of $Mn_3Sn$ layer thickness.

### III. Longitudinal resistivity and anomalous Hall effect of $Mn_3Sn$ thin film

Figure S6(a) shows zero-field longitudinal resistivity $(\rho_{xx})$ as a function of temperature over the temperature range 5 K to 400 K for the 12 nm $Mn_3Sn$ film. It exhibits a metallic behaviour with a residual resistivity ratio (RRR) ~ 1.6 which manifests a high-quality thin film. Note that, Ru resistivity is subtracted to calculate the $\rho_{xx}$ for $Mn_3Sn$ film using a parallel resistor model. We have also investigated Hall resistivity $(\rho_{xy})$ as a function of out-of-plane magnetic field ($H_z$ // (0001)) at room temperature for the same film (Fig. S6(b)). As expected from symmetry when $H_z$ // (0001) it does not show anomalous Hall effect[7].

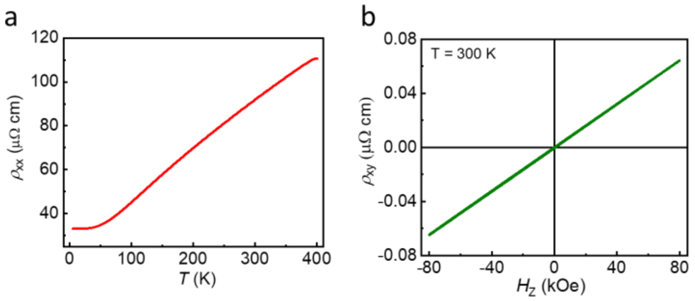


**Fig. S6. Longitudinal and anomalous Hall resistivity of $Mn_3Sn$.** (a) Temperature dependence of $\rho_{xx}$ and (b) $\rho_{xy}$ as a function of out-of-plane magnetic field ($H_z$ // (0001)) at room temperature for the 12 nm $Mn_3Sn$ film.

### IV. Effective magnetization and Gilbert damping constant in $Mn_3Sn$/Py films

ST-FMR d.c. voltage, $V_{mix}$ at $\varphi$ ~ 45º for the different frequencies ($f$ = 4 - 14 GHz) are shown in Fig. S7(a) for $Mn_3Sn$ (12 nm)/Py(5 nm) structure. $V_{mix}$ are fitted using Eq.1. (Main text) and, extracted $H_{res}$ and $\Delta H$. Frequency ($f$) as a function of $H_{res}$ is fitted (Fig. S7(b)) using the Kittel formula (Eq. S1) to calculate the effective magnetization ($M_{eff}$). Similarly, $\Delta H$ as a function of '$f$' is fitted (Fig. S7(c)) using Eq. S2 to extract the Gilbert damping constant ($\alpha$). $M_{eff}$ and $\alpha$ are 774 emu/cm$^3$ and 0.016 respectively for $Mn_3Sn$ (12 nm)/Py(5 nm) structure. Note that $M_{eff}$ and $\alpha$ are independent of the thickness ($d_{AFM}$) of the $Mn_3Sn$ layer (Inset of Fig. S7(b-c)).

$$f = \left(\frac{\gamma}{2\pi}\right)[H_{res}(H_{res} + 4\pi M_{eff})]^{1/2} \quad \text{(S1)}, \qquad \Delta H = \Delta H_0 + \left(\frac{2\pi\alpha}{\gamma}\right) f \quad \text{(S2)}$$

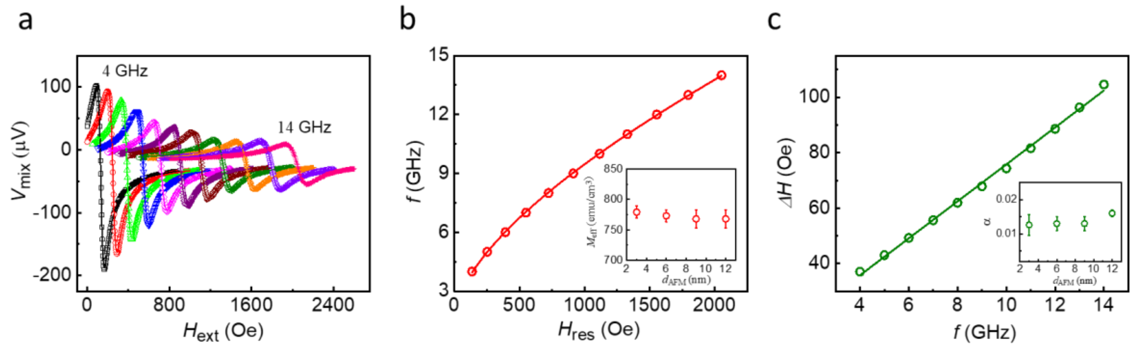


**Fig. S7. ST-FMR data of $Mn_3Sn$/Py.** (a) Frequency dependence of $V_{mix}$ along with the fits for $Mn_3Sn$ (12 nm)/Py(5 nm) (b) '$f$' as a function of $H_{res}$ for the same sample. Inset shows thickness dependence of $M_{eff}$. (c) $\Delta H$ as a function of '$f$' for the same sample. Inset shows the variation of 'α' with $d_{AFM}$.

## V. Angular variation of $V_S$ and $V_A$ for different thicknesses of $Mn_3Sn$ layer

The $V_S(\varphi)$ and $V_A(\varphi)$ for the $Mn_3Sn$($d_{AFM}$ = 3-9 nm)/Py(5 nm) structures show similar angular dependence (Fig. S8(a-f)) like $Mn_3Sn$(12 nm)/Py(5 nm) film (Fig. 3(a-b) in main text). These results show that $\tau_{z,AD}$, $\tau_{z,FL}$ do not depend on $d_{AFM}$. Also, the addition of spin-pumping contribution, which shows $\sin(\varphi)$ angular dependence[8], to $\tau_{y,AD}$ does not reproduce $V_S(\varphi)$. This rules out the spin-pumping contribution for all the films. The asymmetric angular variation of $V_A$ further confirms the presence of $p_z$. The spin-torque

efficiency[9-10] due to $p_y$ ($\theta_y$) and $p_z$ ($\theta_z$) as a function of $d_{AFM}$ are shown in fig. S8 (g,h). Both $\theta_y$ and $\theta_z$ show a weak $d_{AFM}$ dependence.

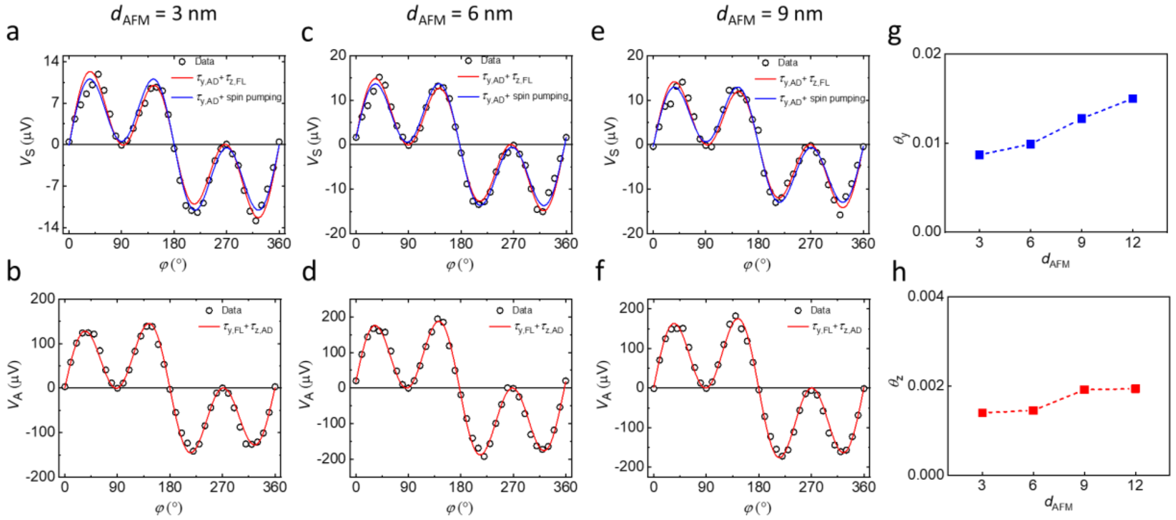


**Fig. S8. Angular dependence of $V_S$ and $V_A$ for pristine Mn3Sn/Py**. Angular dependence of $V_S$ and $V_A$ along with fits for 3 nm (a,b), 6 nm (c,d) and 9 nm (e-f) Mn3Sn layers with Py layer on top for the 0° device $(I_{RF} \parallel [01\bar{1}0])$. Blue curves are the fits based on spin-pumping contribution and $\tau_{y,AD}$. (g-h) Spin-torque efficiency due to $p_y$ ($\theta_y$) and $p_z$ ($\theta_z$) as a function of $d_{AFM}$.

## VI. Theory and spin Hall conductivity tensor for Mn3Sn

In linear response theory, the spin current $\boldsymbol{j}^\gamma$ with spin polarization along $\gamma$ induced by the electric field $\boldsymbol{E}$ is given as

$$j_\alpha^\gamma = \sum_{\beta=x,y,z} \sigma_{\alpha\beta}^\gamma E_\beta,$$

where $\sigma_{\alpha\beta}^\gamma$ $(\alpha,\beta,\gamma = x,y,z)$ is the rank-3 spin conductivity tensor. $\alpha$ corresponds to the components of the spin current and $\beta$ is the direction of the applied electric field. It comprises longitudinal $(\alpha = \beta)$ and transversal $(\alpha \neq \beta)$ spin currents including the SHE. The shape of $\sigma_{\alpha\beta}^\gamma$ is determined by the symmetry of the lattice and the magnetic order by imposing the invariance of $\sigma_{\alpha\beta}^\gamma$ under symmetry operations. It can be decomposed into an odd and an even part $\sigma_{\alpha\beta}^\gamma = \sigma_{\alpha\beta}^{\gamma,\text{odd}} + \sigma_{\alpha\beta}^{\gamma,\text{even}}$, where the former (latter) acquires a changes sign (does not change sign) upon reversing the magnetic order. In the measurement setup, the electric field is

applied along $x$ (0° device) and $y$ (90° device) depending on the device and only the $z$ component of the spin current passes through the interface and exerts a torque on the ferromagnetic layer. Therefore, only the elements $\sigma_{zx}^{\gamma}$ (0° device) and $\sigma_{zy}^{\gamma}$ (90° device) are relevant for the interpretation of our data. Below, we discuss the shape of the spin conductivity tensor for $Mn_3Sn$.

The space group P6$_3$/mmc of the non-collinear antiferromagnet $Mn_3Sn$ has been identified by our XRD measurements. The spontaneous symmetry breaking due to the chiral magnetic order depicted in Fig. 1(e) lowers the symmetries to the magnetic point group (MPG) m'm'm. This MPG possesses three perpendicular (time-reversal) mirror planes, where the mirror plane m (without a time-reversal operation) is perpendicular to the cluster magnetic octupole moment (blue arrow in Fig. 1(e), upper panel) of the spin texture. The spin conductivity tensor

$$\boldsymbol{\sigma}^x = \begin{pmatrix} \sigma_{xx}^x & 0 & 0 \\ 0 & \sigma_{yy}^x & \sigma_{yz}^x \\ 0 & \sigma_{zy}^x & \sigma_{zz}^x \end{pmatrix}, \boldsymbol{\sigma}^y = \begin{pmatrix} 0 & \sigma_{xy}^y & \sigma_{xz}^y \\ \sigma_{yx}^y & 0 & 0 \\ \sigma_{zx}^y & 0 & 0 \end{pmatrix}, \boldsymbol{\sigma}^z = \begin{pmatrix} 0 & \sigma_{xy}^z & \sigma_{xz}^z \\ \sigma_{yx}^z & 0 & 0 \\ \sigma_{zx}^z & 0 & 0 \end{pmatrix}$$

is given for the cluster magnetic octupole moment parallel to the $x$ axis. Here, red (blue) elements indicate even (odd) behavior under time reversal. If the electric field is applied along $x$, the even spin current through the interface is polarized along $y$ and the odd one is polarized along $z$. The $z$-polarized spin currents $j_z^z = \sigma_{zx}^z |E| \cos(\varphi)$, which correspond to a magnetic SHE, follow a cosine behavior as the electric field is rotated by an in-plane angle $\varphi$ between field direction and $x$ axis. Hence, the $z$ spin polarization originating from the bulk magnetic SHE can be suppressed if $\varphi = \pm 90°$, i.e., it vanishes for the 90° device.

## VII. SOT after setting magnetic domain in the presence of an external magnetic field

To investigate how the large external magnetic field influences $p_{\mathrm{y}}$ and $p_{\mathrm{z}}$, we have

applied $H_{\mathrm{set}}$ = ± 7 T to set a specific spin texture of $Mn_3Sn$ before performing the ST-FMR experiments (Fig. 1e upper panel, main text) and then measured the angular dependence of $V_S$ and $V_A$ of $Mn_3Sn$(12 nm)/Py(5 nm) at 300 K. The angular dependence remains unchanged for 0° device (Fig. S9(a-d)) compared to the pristine films (Fig. 3(a-d), Main text). The strength and sign of the torques are also remain the same after reversing the magnetic field (i.e., reversing the spin texture of $Mn_3Sn$) for a particular device. For example, $\tau'_{\mathrm{y,AD}}$ and $\tau'_{\mathrm{z,AD}}$ are 0.17 (0.16) and 0.022 (0.021) when $H_{\mathrm{set}}$ = + 7 T (-7 T) for the 0° device. This shows that $p_{\mathrm{y}}$ and $p_{\mathrm{z}}$ are even under magnetic field.

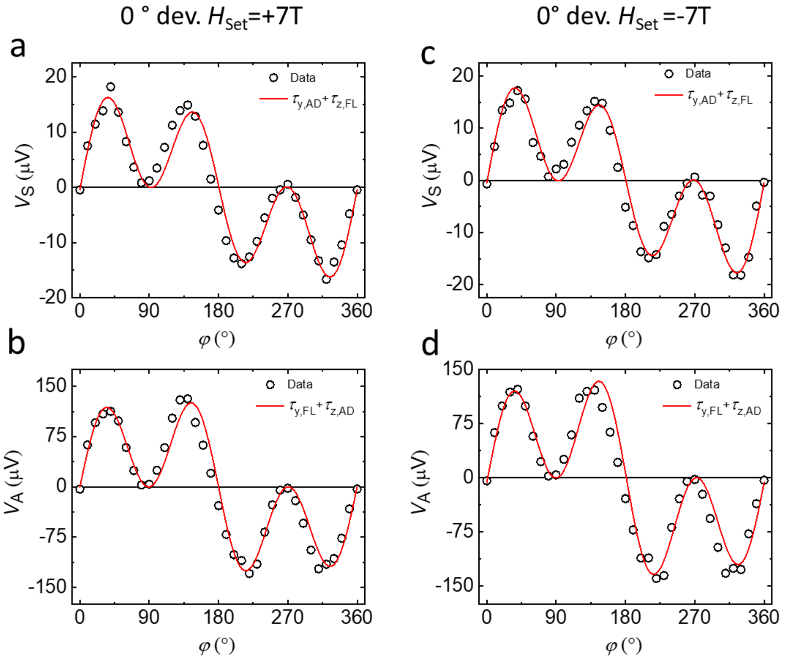


**Fig. S9. Angular dependence of $V_S$ and $V_A$ in $Mn_3Sn$/Py.** (a-d) Angular dependence of $V_S$ and $V_A$ for $Mn_3Sn$(12 nm)/Py(5 nm) after applying an external in-plane magnetic field $H_{\mathrm{set}}$ = ± 7T at 300 K for 0° device.

## VIII. Spin-orbit torques for different crystallographic directions of $Mn_3Sn$

We have measured SOTs for φ = 0°, 45° and 90°. Note that $\varphi = 0°$ represents the device where $I_{\mathrm{RF}}$ is along the in-plane crystallographic direction $[01\bar{1}0]$ of $Mn_3Sn$. The torque due to $p_{\mathrm{y}}$ ($\tau'_{\mathrm{y,AD}}$) is independent of the crystal orientation/in-plane device angle whereas torques due to $p_{\mathrm{z}}$ ($\tau'_{\mathrm{z,AD}}$ and $\tau'_{\mathrm{z,FL}}$) show a small angular dependence (Fig. S10 (a-c)).

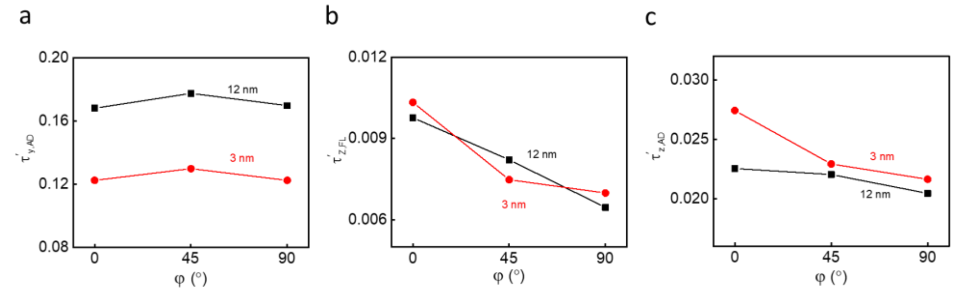


**Fig. S10. Crystallographic dependence of SOTs in Mn3Sn/Py.** (a-c) $\tau'_{y,AD}$, $\tau'_{z,AD}$ and $\tau'_{z,FL}$ as a function of φ for two different thicknesses of $Mn_3Sn$.

## IX. Line-shape, $V_S(\varphi)$, $V_A(\varphi)$ in Cu/Py

$V_{mix}$ at $\varphi$ = 45° for different frequencies ($f$ = 4 - 14 GHz) are shown in Fig. S11(a) for Cu(5 nm)/Py(5 nm) film. $V_{mix}$ are fitted using Eq.1. and extracted $H_{res}$ and $\Delta H$. Frequency ($f$) as a function of $H_{res}$ is fitted (Fig. S11(b)) using the Kittel formula (Eq. S1) to calculate the effective magnetization ($M_{eff}$). $\Delta H$ as a function of '$f$' is fitted (Fig. S11(c)) using Eq. S2 to extract the Gilbert damping constant ($\alpha$). $M_{eff}$ and $\alpha$ are 843 emu/cm$^3$ and 0.006 respectively. $V_S(\varphi)$ and $V_A(\varphi)$ show the presence of $\tau_{y,AD}$, $\tau_{y,FL}$, $\tau_{z,AD}$ and $\tau_{z,FL}$ (Fig. S11(d-e)).

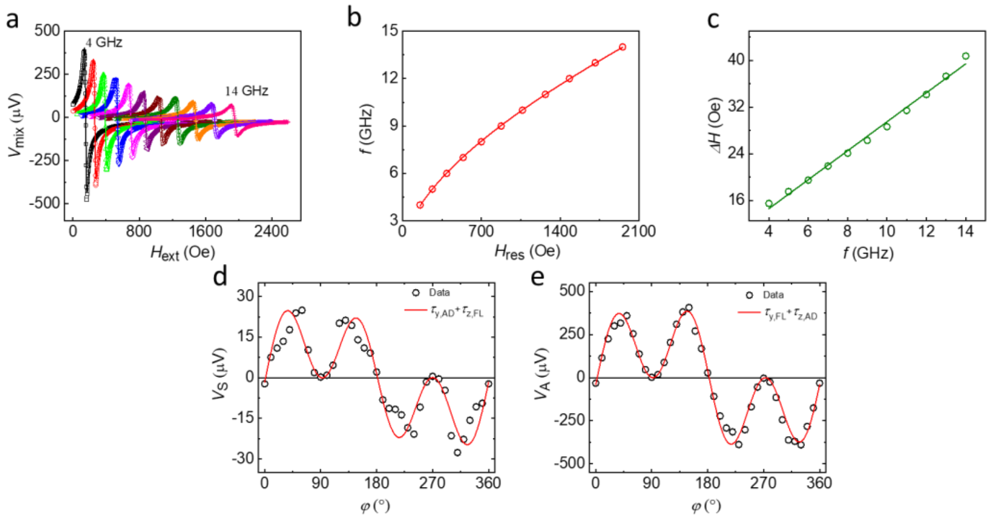


**Fig. S11. ST-FMR data and analysis of Cu/Py.** (a) Frequency dependence of $V_{mix}$ along with the fits for the Cu(5 nm)/Py(5 nm). (b) '$f$' as a function of $H_{res}$. (c) $\Delta H$ as a function of '$f$'. (d-e) Angular dependence of $V_S$ and $V_A$ at 300 K.

## X. Line-shape, $V_S(\varphi)$, $V_A(\varphi)$ in Ru/Py

$V_{mix}$ at $\varphi$ = 45° for different frequencies ($f$ = 4 - 14 GHz) are displayed in Fig. S12(a) for Ru(5 nm)/Py(5 nm) film. Using Eq.1, $V_{mix}$ are fitted and, $H_{res}$ and $\Delta H$ are extracted as fit parameters. Frequency ($f$) as a function of $H_{res}$ is fitted (Fig. S12(b)) using the Kittel formula (Eq. S1) to calculate the effective magnetization ($M_{eff}$). $\Delta H$ as a function of '$f$' is fitted (Fig. S12(c)) using Eq. S2 to extract the Gilbert damping constant ($\alpha$). $M_{eff}$ and $\alpha$ are 887 emu/cm$^3$ and 0.007 respectively. Here also, $V_S(\varphi)$ and $V_A(\varphi)$ show the presence of $\tau_{y,AD}$, $\tau_{y,FL}$, $\tau_{z,AD}$ and $\tau_{z,FL}$ (Fig. S12(d-e)) and angular variation shows a sign change as spin Hall angle of Ru is opposite to Cu or Pt.

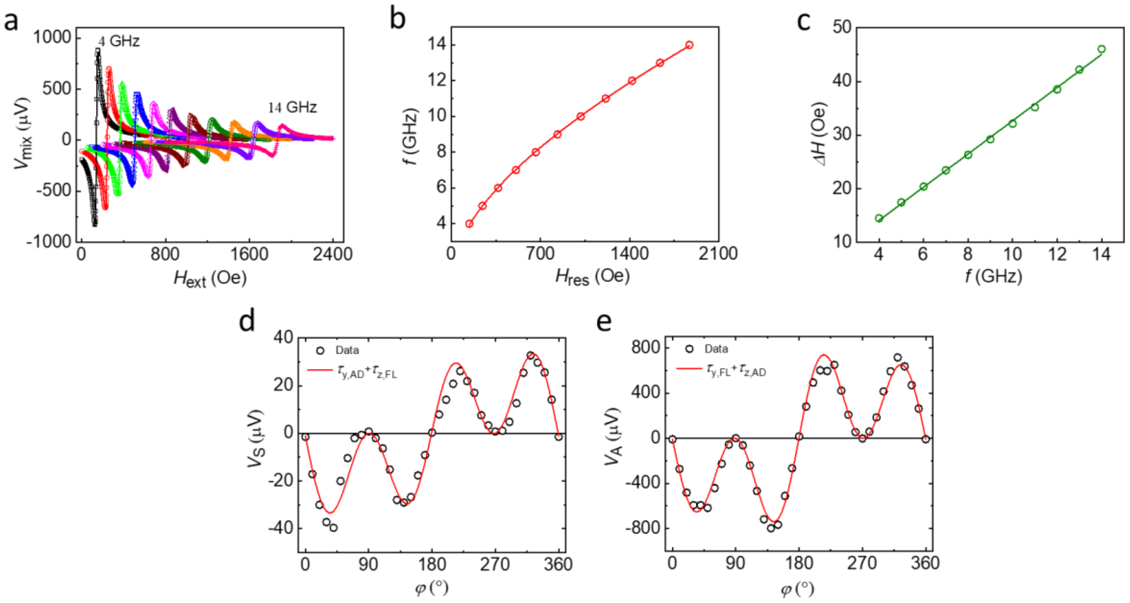


**Fig. S12. ST-FMR data and analysis of Ru/Py.** (a) Frequency dependence of $V_{mix}$ along with the fits for the Ru(5 nm)/Py(5 nm). (b) '$f$' as. a function of $H_{res}$. (c) $\Delta H$ as a function of '$f$'. (d-e) Angular dependence of $V_S$ and $V_A$ at 300 K.

## XI. Line-shape, $V_S(\varphi)$, $V_A(\varphi)$ and in Re/Py

$V_{mix}$ at $\varphi$ = 45° for different frequencies ($f$ = 4 - 14 GHz) are shown in Fig. S13(a) for Re(5 nm)/Py(5 nm) film. $V_{mix}$ are fitted using Eq.1. and extracted $H_{res}$ and $\Delta H$. Frequency ($f$) as a function of $H_{res}$ is fitted (Fig. S13 (b)) using the Kittel formula (Eq. S1) to

calculate the effective magnetization ($M_{\mathrm{eff}}$). $\Delta H$ as a function of '$f$' is fitted (Fig. S13 (c)) using Eq. S2 to extract the Gilbert damping factor ($\alpha$). $M_{\mathrm{eff}}$ and $\alpha$ are 835 emu/cm$^3$ and 0.012 respectively. $V_{\mathrm{S}}(\varphi)$ and $V_{\mathrm{A}}(\varphi)$ show the presence $\tau_{\mathrm{y,AD}}$, $\tau_{\mathrm{y,FL}}$, $\tau_{\mathrm{z,AD}}$ and $\tau_{\mathrm{z,FL}}$ (Fig. S13(d-e)) and angular variation shows a sign change as spin Hall angle of Re is opposite to Cu or Pt.

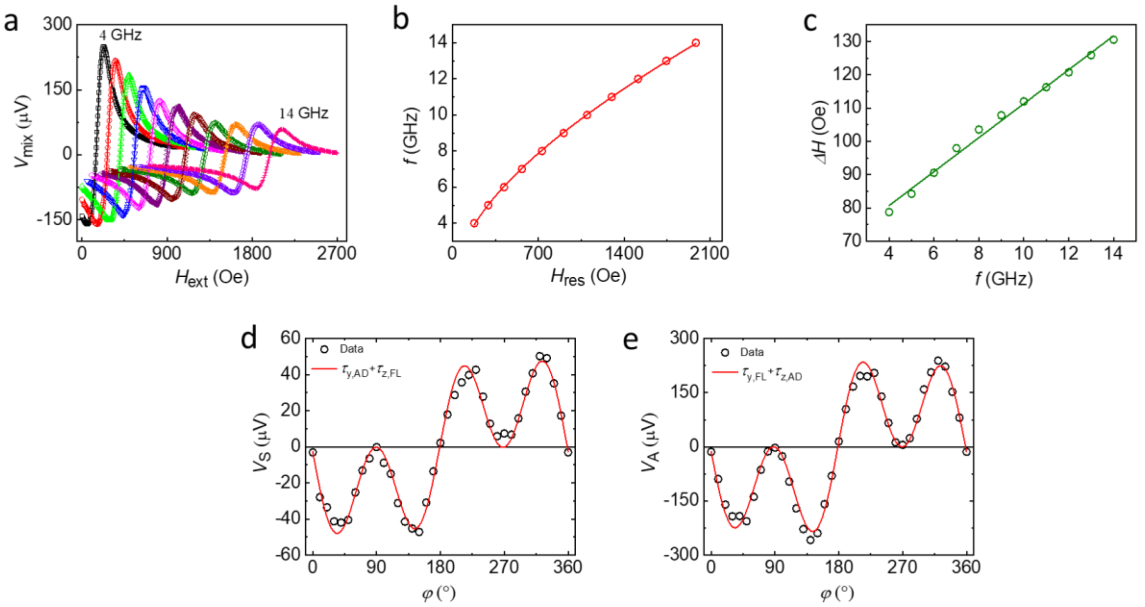


**Fig. S13. ST-FMR data and analysis of Re/Py.** (a) Frequency dependence of $V_{\mathrm{mix}}$ along with the fits for the Re(5 nm)/Py(5 nm). (b) '$f$' as. a function of $H_{\mathrm{res}}$. (c) $\Delta H$ as a function of '$f$'. (d-e) Angular dependence of $V_{\mathrm{S}}$ and $V_{\mathrm{A}}$ at 300 K.

**XII. Effect of Cu and Pt insertion in between $Mn_3Sn$ and Py**

Here, we have investigated the SOT after inserting 2 nm Cu in between $Mn_3Sn$ and Py layer. This helps to break the interface between $Mn_3Sn$ and Py. The angular dependence of $V_{\mathrm{S}}$ and $V_{\mathrm{A}}$ for 0° device in $Mn_3Sn$(12 nm)/Cu(2 nm)/Py(5 nm) shows similar behavior (Fig. S14(a-b)) like $Mn_3Sn$(12 nm)/Py(5 nm) film (Fig. 3(a-b), Main text) which shows a robust $p_{\mathrm{z}}$ after Cu insertion too. Further, we have measured the SOTs for 0° device after inserting 2 nm Pt in between $Mn_3Sn$ and Py layer which also shows the presence of $\tau_{\mathrm{y,AD}}$, $\tau_{\mathrm{y,FL}}$, $\tau_{\mathrm{z,AD}}$ and $\tau_{\mathrm{z,FL}}$ (Fig. S14(c-d)). The increase in the magnitude of $V_{\mathrm{S}}$ and $V_{\mathrm{A}}$ originates from the finite $\tau_{\mathrm{y,AD}}$ and $\tau_{\mathrm{y,FL}}$ of the Pt layer. This clearly shows that the interface near to Py is important

for the generation of $p_z$. The magnitudes of $\tau'_{y,AD}$, $\tau'_{z,AD}$, $\tau'_{z,FL}$ are 0.12 (0.28), 0.023 (0.031), 0.026 (0.04) for Cu (Pt) insertion, respectively.

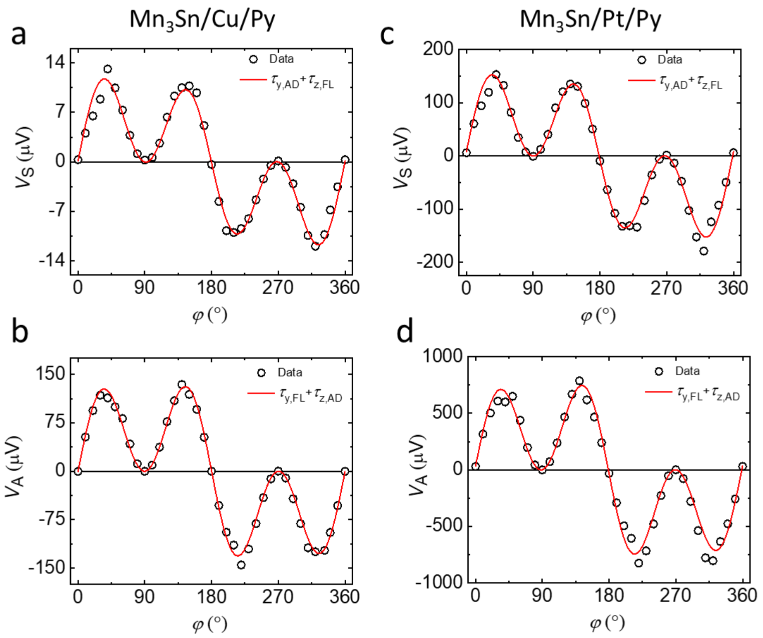


**Fig. S14. $V_S(\varphi)$ and $V_A(\varphi)$ in Mn3Sn/Cu/Py and Mn3Sn/Pt/Py.** Angular dependence of $V_S$ and $V_A$ at 300 K along with fits for (a-b) Mn3Sn/Cu/Py and (c-d) Mn3Sn/Pt/Py structures.

## XIII. Spin-orbit torque in poly-crystalline $Mn_3Sn$ films

We have grown polycrystalline 20 nm thick $Mn_3Sn$ on $Al_2O_3$ substrate without a Ru buffer layer. The anti-damping ($\tau_{y,AD}$) torque due to $p_y$ is not present and $V_S(\varphi)$ shows a very unusual angular variation (Fig. S15a). It is worth noting here that the epitaxial $Mn_3Sn$ (0002) film does not show an AHE whereas polycrystalline $Mn_3Sn$ exhibits a large AHE which makes the analysis more complicated. On the other hand, the angular dependence of $V_A$ indicates the presence of a $\tau_{z,AD}$ ($\tau'_{z,AD} =$ 0.028) due to $p_z$ and Oersted field dominating field-like torque (Fig. S15b). We believe that there are two reasons which might affect the $p_z$. Firstly, from structural analysis, we find that the magnetic properties of Py films grown on polycrystalline $Mn_3Sn$ are different, e.g higher coercivity. Secondly, we find that the interface roughness increases significantly for growth on polycrystalline $Mn_3Sn$ thin films as compared to epitaxial

Mn3Sn thin films.

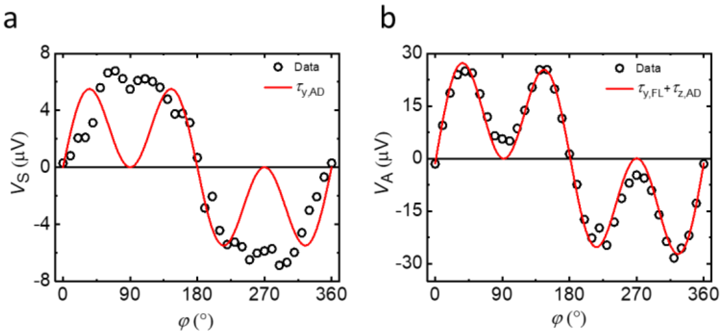


**Fig. S15. $V_S(\varphi)$ and $V_A(\varphi)$ in poly-crystalline Mn3Sn films.** (a-b) Angular dependence of $V_S$ and $V_A$ at 300 K for polycrystalline Mn3Sn film.

## XIV. SOTs in Cu/Py and Pt/Py bilayers on Si/SiO2 and Al2O3

Angular dependence of $V_S$ and $V_A$ for Cu(5 nm)/Py(5 nm) and Pt(5 nm)/Py(5 nm) bilayers which are grown on Si(001)/SiO2(25 nm) substrate shows that $\tau_{z,AD}$ and $\tau_{y,FL}$ (Fig. S16(a,b,e,f) are very small compared to the same structures grown on Al2O3(0001) substrate (Fig. S16(c,d,g,h). The better crystallinity of Cu/Py and Pt/Py on Al2O3 substrate which is evident from XRD (Fig. S3), is responsible for the large $\tau_{z,AD}$ and $\tau_{z,FL}$. This clearly demonstrates that the spin-swapping mechanism dominates when the disorder is less.

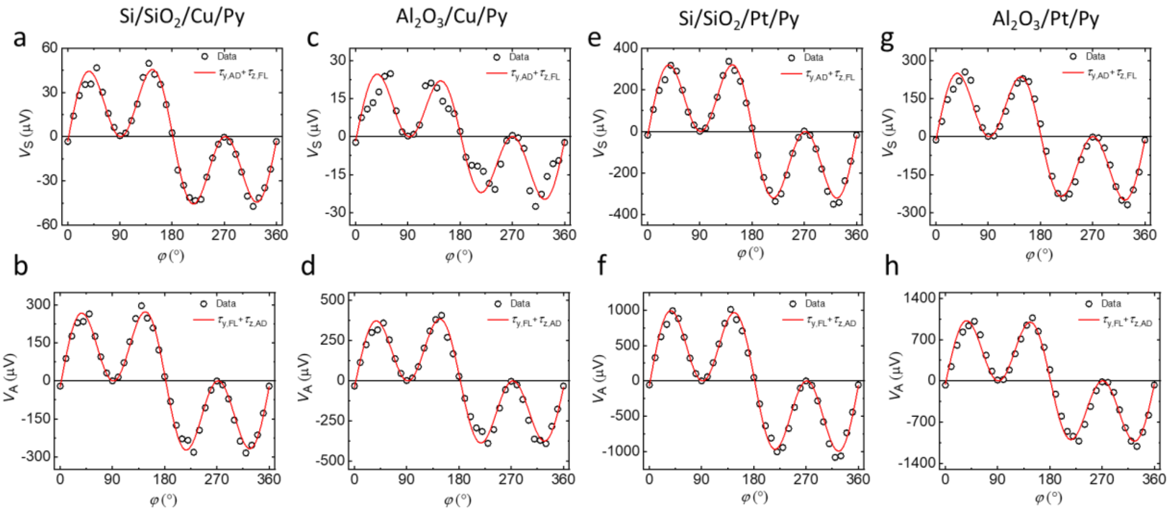


**Fig. S16. $V_S(\varphi)$ and $V_A(\varphi)$ for Cu/Py and Pt/Py.** Angular variation of $V_S$ and $V_A$ for Cu/Py and Pt/Py grown on Si/SiO2 (a,b,e and f) and Al2O3 (c,d,g and h) substrates.

## XV. Comparison of SOTs in GSG and GS-type device

The shape and magnitude of $V_S(\varphi)$ and $V_A(\varphi)$ are strongly depends on the device geometry. Besides torques due to $p_y$, we observed a finite $\tau_{z,FL}$ and $\tau_{z,AD}$ torques in GSG device whereas only significant $\tau_{z,FL}$ dominated in GS devices[11]. A comparison of $V_S(\varphi)$ and $V_A(\varphi)$ from GS and GSG-type devices are shown in Fig.S17. For the 12 nm $Mn_3Sn$/Py films $\tau'_{y,AD}$ and $\tau'_{z,FL}$ are 0.16 (0.17) and 0.15 (0.01) for GS-type (GSG-type) device, respectively.

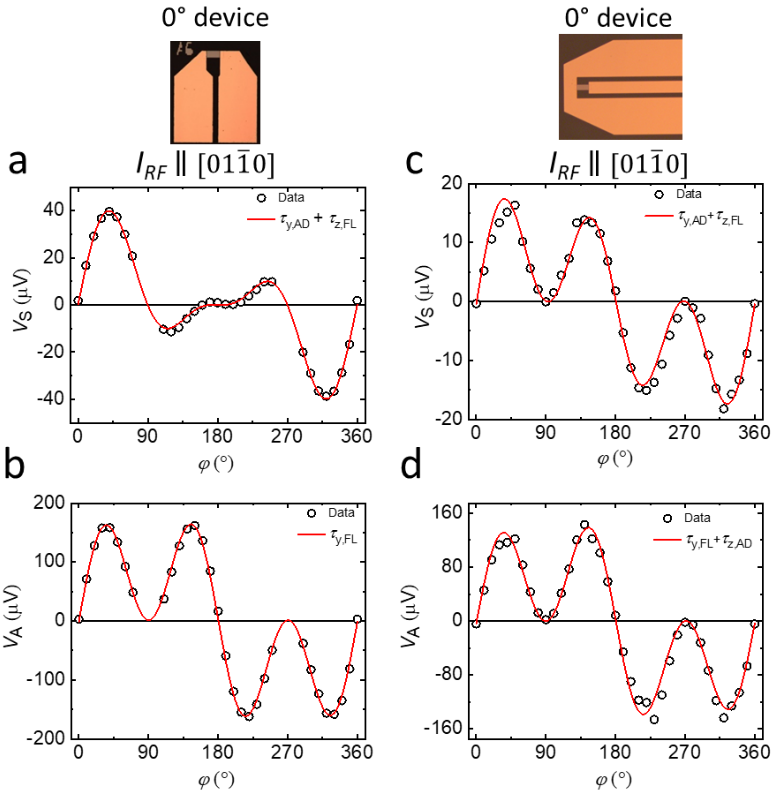


**Fig. S17. $V_S(\varphi)$ and $V_A(\varphi)$ in GSG and GS-type device.** Angular variation of $V_S$ and $V_A$ for $Mn_3Sn$(12 nm)/Py(5 nm) measured with GS-type (a-b) and GSG-type (c-d) devices.

## XVI. Spin-orbit torques in Cu/Fe and Cu/Ni bilayers

The theoretical[12] and experimental[13] developments of the orbital Hall effect (OHE) show only the existence of a $p_y$ polarization which strongly depends on the magnetization of the ferromagnets. If OHE was the mechanism in our devices then $p_z$ should strongly depend on the magnetization of the ferromagnet. Thus, we have made similar devices of Cu/Py where the Py layer was replaced by Fe or Ni. However, we see a very weak magnetization dependence of $p_z$ (reduced by half from Cu/Fe to Cu/Ni) although we do find that $p_y$ (reduced 4 times from Cu/Fe to Cu/Ni) strongly depends on the magnetization (see Fig. S18). This rules out the OHE as the origin of $p_z$ and supports the spin-swapping origin of $p_z$. Note that in spin swapping mechanism, $p_z$ weakly depends on the magnetization of the ferromagnets[14].

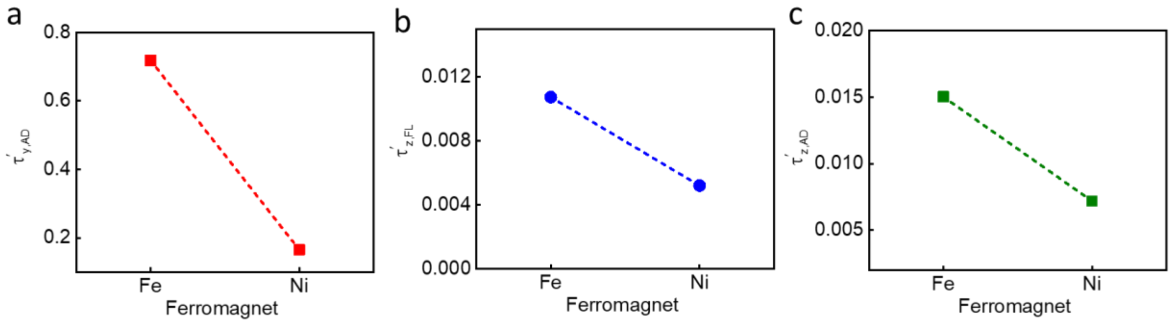


**Fig. S18. Magnetization dependence of SOTs in Cu/Fe and Cu/Ni bilayers.** $\tau'_{y,AD}$, $\tau'_{z,AD}$ and $\tau'_{z,FL}$ for Cu/Ni and Cu/Fe bilayers.

## References


[1] Vlieg, E. and Lohmeier M. Angle Calculations for a Six-Circle Surface X-ray Diffractometer, *J. Appl. Cryst.* **26**, 706-716 (1993).

[2] Schamper, C. Meyerheim, H. L.; and  Moritz, W. Resolution correction for surface X-ray diffraction at high beam exit angles, *J. Appl. Cryst.* **26**, 687-696 (1993).

[3] E. Vlieg. Integrated Intensities Using a Six-Circle Surface X-ray Diffractometer, *J. Appl.*

*Cryst*. **30**, 532-543 (1997).

[4] Gates-Rector, S. D. and Blanton, T. N, The Powder Diffraction File: A Quality Materials Characterization Database, Powder Diffr. **34**, 352-360 (2019).

[5] W. F. Kuhs, Generalized Atomic Displacements in Crystallographic Structure Analysis, *Acta Cryst.* **A48**, 80-98 (1992).

[6] Markou, A. et al. *Phys. Rev. Mater.* **2**, 051001 (2018).

[7] S. Nakatsuji, N. Kiyohara, T. Higo, *Nature* **527**, 212-215 (2015).

[8] Sklenar, J. *et al.* Driving and detecting ferromagnetic resonance in insulators with the spin Hall effect, *Phys. Rev. B* **92**, 174406 (2015).

[9] Nan, T. et al. Controlling spin current polarization through non-collinearantiferromagnetism. Nat. Commun. 11, 4671 (2020).

[10] You, Y. *et al.* Cluster magnetic octupole induced out-of-plane spin polarization in antiperovskite antiferromagnet. *Nat. Commun.* **12**, 6524 (2021).

[11] Horaguchi *et. al.* Highly accurate evaluation of spin-torque efficiency by measuring in-plane angular dependence of spin-torque ferromagnetic resonance, J. Magn. Magn. Mater. 505 166727 (2020).

[12] Go, D., Jo, D., Kim, C. & Lee, H.-W. Intrinsic Spin and Orbital Hall Effects from Orbital Texture. *Phys. Rev. Lett.* **121**, 086602 (2018).

[13] Lee, D. *et al.* Orbital torque in magnetic bilayers. *Nat. Commun.* **12**, 6710 (2021).

[14] Park, H.-J. et al. Spin Swapping Effect of Band Structure Origin in Centrosymmetric Ferromagnets. Phys. Rev. Lett. 129, 037202 (2022).
